# Transcription factor A, mitochondrial promotes lymph node metastasis and lymphangiogenesis in epithelial ovarian carcinoma

**DOI:** 10.1515/med-2024-1089

**Published:** 2025-02-07

**Authors:** Reziwanguli Wubuli, Mayinuer Niyazi, Lili Han, Mayinuer Aierken, Lingling Fan

**Affiliations:** Graduate School of Xinjiang Medical University, Urumqi, 830001, China; Department of Gynecology, People’s Hospital of Xinjiang Uygur Autonomous Region, Urumqi, China

**Keywords:** epithelial ovarian cancer, TFAM, epithelial–mesenchymal transition, lymphatic metastasis, lymphangiogenesis

## Abstract

**Background:**

Mitochondria play a central, multifunctional role in cancer progression. However, the mechanism of mitochondrial genes in epithelial ovarian cancer (EOC) remains unclear. This study aimed to screen candidate mitochondrial genes in EOC and then to investigate their biological functions and potential mechanisms.

**Methods:**

We downloaded Gene Expression Omnibus RNA-seq profiles and identified mitochondrial differentially expressed genes in EOC by bioinformatics analysis. Transcription factor A, mitochondrial (TFAM) expression in EOC tissues was determined by immunohistochemistry. *In vitro* assays were applied to clarify TFAM function in EOC.

**Results:**

The bioinformatics analysis results showed that the mitochondrial genes *TFAM*, *HSPE1*, and *CYC1* were significantly upregulated (*P* < 0.05) in EOC, and their upregulation was associated with a poor prognosis. *TFAM* was highly expressed in EOC tissues and significantly associated with clinical stage (*P* = 0.004), lymph node metastasis (*P* = 0.043), and overall survival (*P* < 0.05). Silencing *TFAM* in EOC cells significantly inhibited cell proliferation and migration and induced cell apoptosis (*P* < 0.05).

**Conclusion:**

TFAM promotes EOC cell secretion of VEGF-A, VEGF-C, VEGF-D, lymphangiogenesis, and EOC lymph node metastasis. Our results may provide new insights into the biological functions and potential mechanisms of TFAM in EOC, which might provide new targets for EOC diagnosis and treatment.

## 1 Introduction

Among gynecological malignancies, ovarian cancer ranks third in terms of incidence but first in terms of mortality rate [1]. Epithelial ovarian cancer (EOC) is the most common (∼90%) pathological type of OC [2,3]. Approximately 75% of all patients with EOC are diagnosed during the late stages, with a relative 5-year survival rate of 29%. EOCs commonly spread via the lymphatic system [4]. As lymphatic node (LN) metastasis is an independent risk factor for EOC treatment resistance, which results in reduced patient survival [5], the molecular mechanisms underlying LN metastasis in EOC have attracted much attention from researchers and clinicians.

Tumor lymphangiogenesis is the first stage of LN metastasis, followed by tumor cell dissemination to the lymphatic vessels, tumor cell transfer to LNs through the lymphatic system, cell seeding, colonization, and growth in LNs [6] Vascular endothelial growth factor (VEGF-C) and VEGF-D as well as their cognate receptor *VEGF-R3* have been reported to be crucial for the onset of lymphangiogenesis [7]. To date, no effective therapeutic approaches have been clinically proposed to inhibit lymphangiogenesis and lymphatic metastasis [8]. However, delving into the regulatory mechanisms governing lymphangiogenesis and lymph node metastasis in EOC could aid in pinpointing targets for therapies that can prevent lymph node metastasis.

Epithelial–mesenchymal transition (EMT) is a process in which epithelial cells acquire mesenchymal features. EMT is associated with various tumor functions, including tumor initiation, malignant progression, tumor stemness, tumor cell migration, intravasation, metastasis, and resistance to therapy [9]. Accumulating evidence suggests that the metastatic ability of EOC cells is facilitated by EMT [10], and EMT plays an important role in EOC progression and spreading. Nevertheless, the molecular mechanism underlying this transition remains poorly understood [11]. Cancer cells, particularly during EMT, fine-tune their metabolic circuitry to meet the increased bioenergetic demands of encountering complex challenges during cellular transit. Therefore, understanding the switch in metabolic pathways during EMT would unravel key vulnerable therapeutic targets [12].

Mitochondria are intracellular organelles that produce most of the energy, metabolites, and reactive oxygen species. These functions also determine the crucial role of mitochondria in cancer progression-related processes, including metastasis.

Based on the aforementioned information, this study aimed to screen candidate mitochondrial genes in EOC and investigate their biological functions and potential mechanisms. According to bioinformatics analysis and *in vivo* gene function experiments, this study revealed that *TFAM* is associated with LN metastasis and a poor prognosis in EOC. *TFAM* promotes tumor-associated lymphangiogenesis and EMT. Consequently, *TFAM* might provide new insights into the pathogenesis of EOC and may represent a therapeutic target.

## 2 Materials and methods

### 2.1 Data sources and bioinformatics analysis

The gene expression dataset GSE209964 [13] for high-grade serous ovarian cancer was obtained from the Gene Expression Omnibus (GEO) database. We performed normalization and differential gene expression analysis for transcriptome count data from 81 primary tumor samples and 7 normal fallopian tube samples using the “DESeq2” R package [14]. We filtered differentially expressed genes (DEGs) with a |logFC| > 2 and an adjusted *P* < 0.05. Volcano plots were generated with ggplot2 and the heatmap package in *R* studio software. DAVID 6.8 was used for gene ontology (GO) and KEGG pathway enrichment analysis of the DEGs and *P* < 0.05 was considered statistically significant [15]. The results were visualized with an online bioinformatics tool (http://www.bioinformatics.com.cn) [16]. Kaplan–Meier plotter (http://kmplot.com/analysis) is a website that enables online validation of survival biomarkers and analyzes the overall survival (OS) of patients with high and low expression of certain genes [17]. The protein expression data of transcription factor A, mitochondrial (TFAM) and its clinical correlations in ovarian cancer were collected from the Clinical Proteomic Tumor Analysis Consortium (CPTAC) database [18]. Validation of the correlations between *TFAM* and *LYVE1*, *PDPN*, and *PROX1* was conducted using the Gene Expression Profiling Interactive Analysis (GEPIA 2) database [16].

### 2.2 Collection of clinical specimens

The experimental tissue samples included 48 ovarian tissue paraffin samples from EOC patients who were hospitalized in the Department of Gynecology, People’s Hospital of Xinjiang Uygur Autonomous Region from May 2008 to December 2013, as well as 38 benign tissue paraffin samples from patients with benign ovarian cysts during the same period. All of these samples were examined by experienced pathologists who confirmed the diagnoses. The age of the patients in the EOC group ranged from 30 to 77 years, with an average of 54.3 ± 12.2 years, and the age of the patients with benign ovarian tumors ranged from 27 to 71 years, with an average of 48.8 ± 15.3 years. The EOC specimens were classified according to the FIGO Staging System for Ovarian Cancer (2014). In the EOC group, 6 cases were in stage I, 9 cases in stage II, 26 cases in stage III, and 7 cases in stage IV.

The EOC patients were followed up from May 2008 and ended in January 2018, with follow-up periods ranging from 4 to 112 months. However, some patients were lost to follow-up due to various reasons, and their data were not included in the survival analysis of this study; the final follow-up rate was 79% (*n* = 38).

### 2.3 Immunohistochemistry

The paraffin-embedded histological section of each serous EOC tissue was 3 μm thick. Tissues were dewaxed, and antigen retrieval was performed using a low pH buffer (pH = 6.0) for 15 min at 100°C. Staining of TFAM was performed using TFAM rabbit monoclonal antibody (diluted 1:50; Abcam Co, Cambridge, UK). Expression of TFAM was scored using the following system. The color intensity was divided into four levels, to be specific, no staining was scored as 0, light yellow staining was scored as 1, brown–yellow staining was scored as 2, and dark brown staining was scored as 3. On the other hand, positive cells were counted under 400× magnification and scored as follows based on positive staining rate: less than 5% was scored as 0, 5–25% was 1, 26–50% was scored as 2, 51–75% was scored as 3, and more than 75% was scored as 4. The scores for immunoreactive intensity and positive cell rate were then multiplied, and the final result was as follows: 0–2 was considered negative (−), 3–4 was considered weakly positive (+), 5–8 was considered mildly positive (++), and 9–12 was considered strongly positive (+++). The results were read by two independent observers who made the scoring decision to control for variability.

### 2.4 Cell culture

The human EOC cell line SK-OV-3 was purchased from Procell Life Science Technology (Wuhan, China) and cultured in McCoy’s 5A medium supplemented with 10% fetal bovine serum (FBS), 100 U/mL penicillin, and 100 μg/mL streptomycin (Gibco, Carlsbad, CA). Human lymphatic endothelial cells (HLECs) were purchased from YaJi Biological (Shanghai, China) and cultured in endothelial cell culture medium from YaJi Biological (Shanghai, China) supplemented with 10% FBS, 100 U/mL penicillin, and 100 μg/mL streptomycin (Gibco, Carlsbad, CA) [19]. The cells were incubated in a humidified atmosphere with 5% CO_2_ at 37°C. The morphology of the cells was observed under an inverted microscope, and the medium was changed every 3 days.


**Informed consent:** All patients or patients’ families signed written informed consent forms.
**Ethical approval:** All the experimental procedures were approved by the Ethics Review Committee of People’s Hospital of Xinjiang Uygur Autonomous Region (KY2021042003), and specimen collection, processing, and analysis for the purpose of this study were performed between September 10 and October 2, 2022.

## 3 TFAM-shRNA lentiviral vector construction and transfection

shRNA-mediated *TFAM* knockdown in SK-OV-3 cells was performed. *TFAM*-shRNA (shRNA sequence: GTAAGTTCTTACCTTCGATTT) and NC (negative control)-shRNA (shRNA sequence: TTCTCCGAACGTGTCACGT) were synthesized and annealed to form double-stranded DNA fragments that were cloned into the GV493 hU6-MCS-CBh-gcGFP-IRES-puromycin lentivirus vector and packaged in HEK 293 cells by Gene Chem Biotechnology (Shanghai, China). The cells grown to 80–90% confluence were transfected with NC-shRNA lentivirus, and the fluorescence intensity was observed by an inverted fluorescence microscope every day until 72 h. The fluorescence expression abundance was high, the infection efficiency was approximately 80%, and the corresponding infection conditions and multiplicity of infection (MOI) of the group with good cell growth could be used as the basis for the subsequent infection experiment. The cells used in this experiment were divided into three groups: control group, cell line without lentiviral transfection; shRNA-NC group, cell line with NC-shRNA lentiviral transfection; and *TFAM*-shRNA group, cell line with *TFAM*-shRNA lentiviral transfection. Silencing of *TFAM* mRNA expression was verified using real-time reverse transcription polymerase chain reaction (RT-qPCR) and further confirmed by western blot analysis.

### 3.1 Cell proliferation of SK-OV-3 cells

Changes in cell proliferation were determined by using the Cell Counting Kit-8 (CCK-8) assay kit (TransGen Biotech, Beijing, China). In particular, SK-OV-3 cancer cells were seeded into 96-well plates at a density of 5,000 cells/well in 100 µL of complete medium and cultured at 37°C. Then, the cells were transfected with *TFAM*-shRNA lentiviral vector and NC-shRNA lentiviral vector. Each group had five biological replicates and incubation for 72 h. Then, 100 µL of CCK-8 reagent was added to each well, and the cells were further cultured for 1 h at 37°C. The optical density value (OD450) was measured by using an xMarkTM microplate reader (Bio-Rad, CA, USA). The assay was performed in triplicate and repeated at least three times.

### 3.2 Cell apoptosis assay

Cells were harvested with 0.25% trypsin (Gibco Life Technologies) and then stained using an Annexin V-FITC Apoptosis Detection Kit (MultiSciences, HangZhou, China) according to the manufacturer’s instructions. After staining with 5 µL of Annexin V-PE and 10 µL 7-AAD and incubation for 5 min at room temperature, the cells were analyzed by flow cytometry (Beckman Coulter, Fullerton, CA).

### 3.3 Cell migration assay

Transwell assays were performed to evaluate the cell migration ability using Transwell chamber inserts with a filter pore size of 8 µm in 24-well plates (Corning Inc., Corning, NY, USA). For this assay, 1  ×  10^5^ cells were seeded into the upper chamber with 100 µL of McCoy’s 5A medium without FBS, while 600 µL of medium containing 15% FBS was placed in the bottom chamber. Cells were incubated at 37°C for 72 h. Moreover, cells migrating into the reverse side of the filter were fixed with 4% paraformaldehyde at room temperature for 20 min and then removed from the chamber, rinsed twice with PBS, and then transferred to the chamber to preadded 400 μL of dye at room temperature for 1 min in the pores of Giemsa dye solution A. Solution A was not discarded, and Giemsa dye solution B was added to 800 μL again. After continuing staining for 5 min, the chamber was removed and rinsed twice with PBS. The cells on the surface of the upper chamber were carefully wiped off with a wet cotton swab, and the bottom of the chamber was allowed to face up and dry naturally. The cells were transferred to a glass slide, and the number of migrated cells was observed under an inverted fluorescence microscope (Eclipse TS100-F, Nikon, Tokyo, Japan) and photographed at a magnification of ×200 in five randomly selected fields and counted.

### 3.4 RT-qPCR

Total RNA was extracted from the tissue and SK-OV-3 cells (Procell Life Science Technology, China) with TRIzol reagent according to the manufacturer’s instructions (Thermo Fisher Scientific; Waltham, MA, USA). cDNA was prepared from total isolated RNA using 5× All-In-One RT MasterMix according to the manufacturer’s instructions (ABM Biotechnology Co., Ltd, Nanjing, China). Gene expression was detected using RT-PCR with EvaGreen Express 2× qPCR MasterMix-Low Rox (ABM Biotechnology Co., Ltd, China) on a Real Time PCR instrument 7,500 Fast (Bio-Rad). The primer sequences for RT-qPCR are listed in Table 1. The relative expression of each target gene was calculated using the 2^−ΔΔCt^ method with glyceraldehyde 3-phosphate dehydrogenase (GAPDH) as the housekeeping gene.

**Table 1:** Primer sequences for RT-qPCR

Primer	Sequence (5′–3′)	Product size (bp)
*TFAM*-F	TGATTCACCGCAGGAAAAGC	179
*TFAM*-R	TTGTGCGACGTAGAAGATCC	
*E-cadherin*-F	CGAGAGCTACACGTTCACGG	119
*E-cadherin*-R	GGGTGTCGAGGGAAAAATAGG	
*SNAIL*-F	TCGGAAGCCTAACTACAGCGA	140
*SNAIL*-R	AGATGAGCATTGGCAGCGAG	
*Vimentin*-F	AGTCCACTGAGTACCGGAGAC	98
*Vimentin*-R	CATTTCACGCATCTGGCGTTC	
*VEGF-A*-F	AGGGCAGAATCATCACGAAGT	75
*VEGF-A*-R	AGGGTCTCGATTGGATGGCA	
*VEGF*-C-F	GGCTGGCAACATAACAGAGAA	159
*VEGF*-C-R	CCCCACATCTATACACACCTCC	
*VEGF*-D-F	ATGGACCAGTGAAGCGATCAT	81
*VEGF*-D-R	GTTCCTCCAAACTAGAAGCAGC	
*GAPDH*_F	GGAGCGAGATCCCTCCAAAAT	197
*GAPDH*_R	GGCTGTTGTCATACTTCTCATGG	

F: forward primer; R: reverse primer.

### 3.5 Western blot analysis

Total cellular protein extraction was performed as described previously [20]. Briefly, the protein was extracted from cells using RIPA buffer (Boster, Wu Han, China) containing protease inhibitors and phosphatase inhibitors. After being fully homogenized on ice with a glass homogenizer, the cells were lysed in an ice bath for 60 min and centrifuged at 12,000 rpm at 4°C for 15 min to collect the supernatants, and the protein concentration was measured using the Easy II Protein Quantitative Kit (BCA) (TransGen, Beijing, China). Afterward, the protein samples (30 µg each loading) were separated in 12% sodium dodecyl sulfate–polyacrylamide gel electrophoresis gels and then transferred onto polyvinylidene fluoride membranes (Millipore, Billerica, MA, USA) in an ice bath. For western blotting, the membranes were incubated with rabbit anti-MtTF1/TFAM antibody (antibody dilution ratio: 1:1,000) (Bioss, Beijing, China), rabbit anti-E cadherin antibody (antibody dilution ratio: 1:500) (Bioss, Beijing, China), rabbit anti-vimentin antibody (antibody dilution ratio: 1:400) (Bioss, Beijing, China), and rabbit anti-SNAIL antibody (antibody dilution ratio: 1:400) (Bioss, Beijing, China) at 4°C overnight. The following day, the membranes were subsequently incubated with horseradish peroxidase-conjugated secondary goat anti-mouse or goat anti-rabbit antibody (antibody dilution ratio: 1:5,000) (Abcam, CA, USA) at room temperature for 1 h. Afterward, the protein bands were detected with an enhanced chemiluminescence reagent (Millipore) using the ChemiScope mini Chemiluminescence Imaging System (Clinx, Shang Hai, China) and quantified using ImageJ software (National Institute of Heath, Bethesda, MD, USA) after normalization to the β-actin level.

### 3.6 Cell proliferation of HLECs

The HLEC single cell suspension was inoculated into a 96-well plate (100 μL/well) and incubated for 24 h. After adhering, the cells were incubated with conditioned medium (CM; fresh HLEC culture medium and SKOV-3 cell supernatant [1:1]). After 24 h of intervention, the culture medium was discarded, 100 μL of 10% CCK-8 solution was added to each well, and the plate was incubated for 1 h. Then, the optical density value (OD450) was measured by using an xMarkTM microplate reader (Bio-Rad, CA, USA).

### 3.7 Tube formation assay

HLECs (1 × 10^4^/well) were seeded into a 48-well plate precoated with 300 µL Matrigel (Corning, CA, USA) and cultured in CM (fresh HLEC culture medium and SKOV-3 cell supernatant [1:1]). After 24 h, HLEC tube formation was assessed by microscopy, and each well was photographed. The number of tube branches and total tube lengths were calculated using MacBiophotonics ImageJ software (Bethesda, MD, USA) [25].

### 3.8 ELISA

SK-OV-3 cell supernatants were collected for ELISA detection 72 h after transfection with *TFAM*-shRNA lentivirus. Then, *VEGF-A*, *VEGF-C*, and *VEGF-D* were detected by ELISA kits (*VEGF-A* kit, Multisciences, Hangzhou, China; *VEGF-C* kit and *VEGF-D* kit, CUSABIO, Wuhan, China) according to the manufacturer’s instructions.

### 3.9 Statistical analysis

SPSS 20.0 software was used for statistical analysis. Data are presented as mean ± SD of at least three independent experiments. Statistical analysis of comparisons between two samples was performed using Student’s *t* test. One-way analysis tests were used for statistical comparisons of more than two groups. The *χ* ^2^ test was applied to compare frequencies of categorical variables between the groups. OS was evaluated using the Kaplan‒Meier method. A *P* value of <0.05 was considered to indicate statistical significance.

## 4 Results

### 4.1 Screening of DEGs of EOC

**Figure 1:**
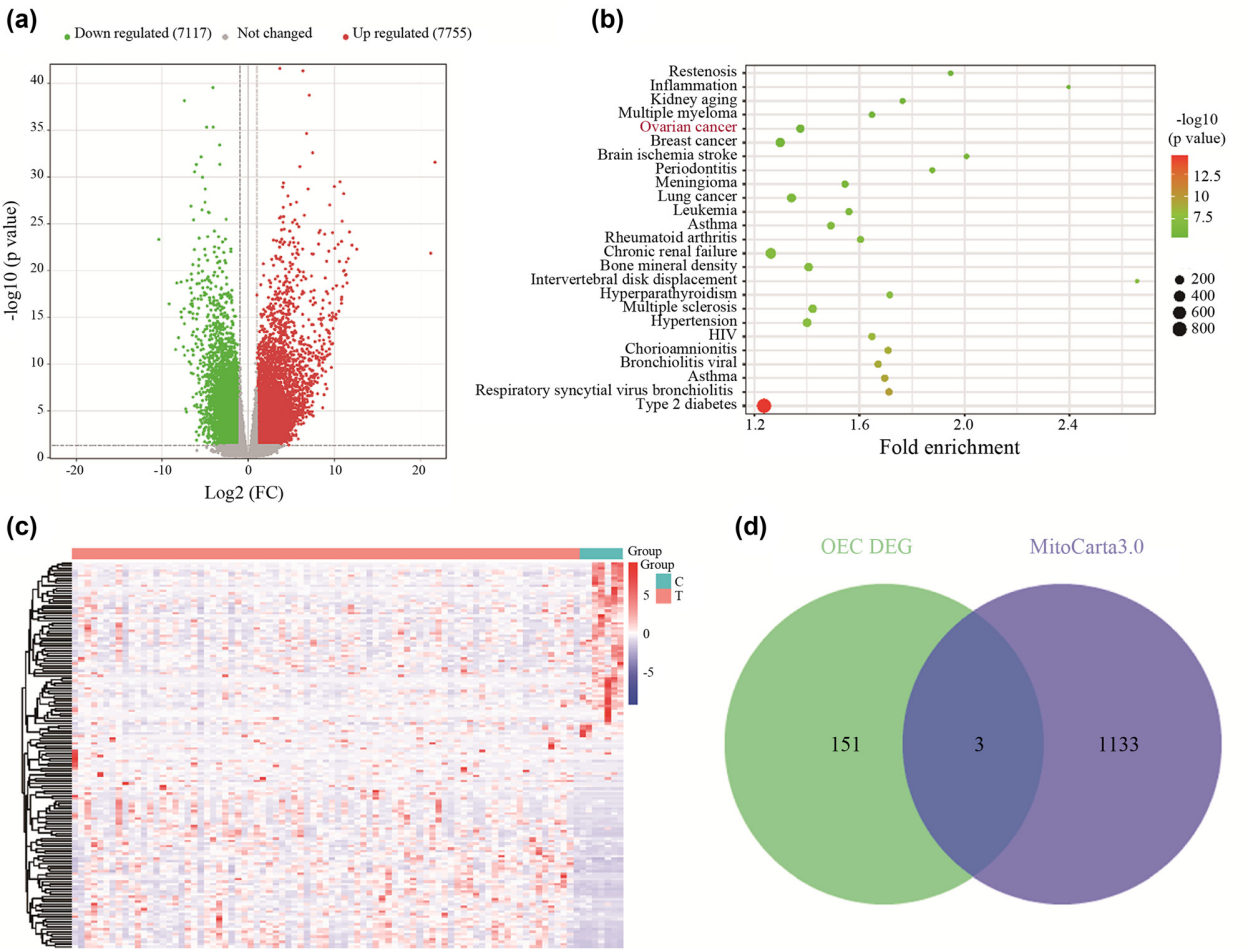
Screening of DEGs in serous ovarian cancer data from the GEO dataset GSE209964. (a) Volcano plot of DEGs. Red indicates upregulated genes and green indicates downregulated genes. (b) Enrichment of ovarian cancer-associated genes by the DAVID online tool. (c) Ovarian cancer-associated DEG cluster analysis and heatmap construction. (d) Venn diagram for intersection of the DEGs related to mitochondria.

We first analyzed the EOC transcriptome sequencing dataset GSE209964 from the GEO database and identified 7,755 upregulated and 7,117 downregulated DEGs (Figure 1a) between 81 high-grade serous ovarian cancer primary tumor tissues and 7 normal fallopian tube tissues. We used the DAVID online tool to enrich ovarian cancer-associated genes and performed cluster analysis and heatmap construction. The results showed that 154 genes were significantly upregulated in ovarian cancer samples versus normal samples (Figure 1b and c). Functional enrichment analysis indicated that the EOC-related genes were mainly involved in GO terms, including cell adhesion, cytoplasmic translation, signal transduction, chemotaxis, plasma membrane, extracellular matrix, integral component of membrane, protein binding, extracellular matrix structural constituent, integrin binding, and receptor binding. Additionally, KEGG pathway enrichment analysis for the EOC-related genes was performed to further study biological function. Briefly, these genes mainly participated in ECM–receptor interaction, cell cycle, cell adhesion molecules, *PI3K-Akt* signaling pathway, chemokine signaling pathway, *MAPK* signaling pathway. To perform further screening of mitochondrial genes, mitochondrial-related gene sets were downloaded from the MitoCarta3.0 database and then overlapped with ovarian cancer-related genes to identify candidate genes. The intersection of the DEGs related to mitochondria is shown in the Venn diagram. Finally, three candidate genes were screened: *TFAM*, *HSPE1*, and *CYC1* (Figure 1d; Table 2).

**Table 2:** Log2 fold change values and *P* values of candidate genes with differential expression between groups

Candidate gene	log2 fold change	*P* value	padj
TFAM	2.409789547	0.004494773	0.016271987
HSPE1	2.028717851	0.001936843	0.008039573
CYC1	5.701148537	0.000619981	0.00308038

### 4.2 mRNA expression of candidate genes in ovarian cancer

**Figure 2:**
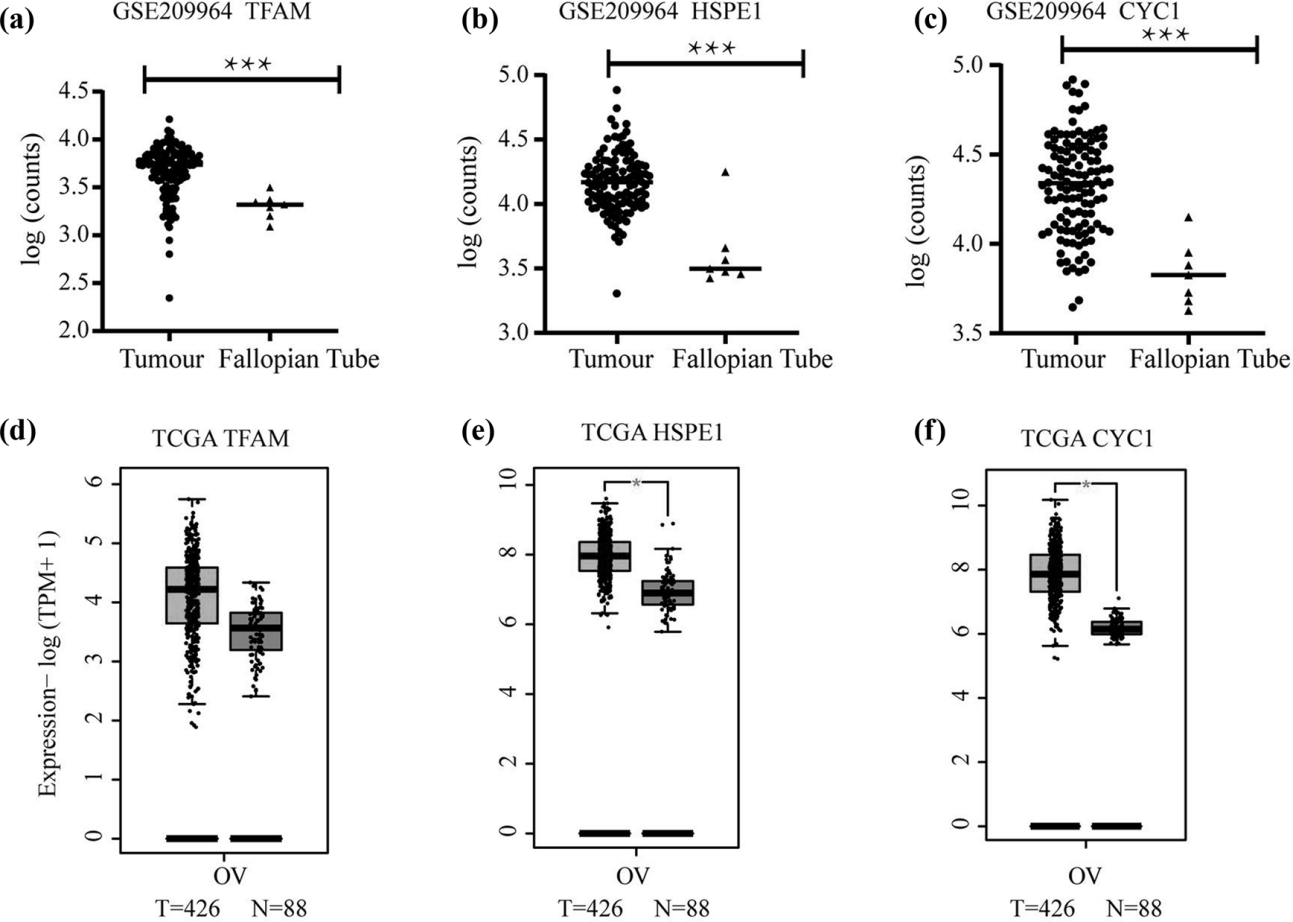
mRNA expression levels of candidate genes in ovarian cancer. (a)–(c) mRNA expression levels of *TFAM*, *HSPE1*, and *CYC1* in high-grade serous ovarian cancer tissue and healthy fallopian tube tissue. (d)–(f) mRNA expression levels of *TFAM*, *HSPE1*, and *CYC1* in ovarian cancer tissue and normal samples in GTEx. ***P < 0.001, *P < 0.05.

To further determine the expression levels of the three candidate genes in ovarian cancer, we further analyzed the expression levels of the three candidate genes in the GSE209964 dataset and the TCGA database, and the analysis results of GSE209964 data showed that the expression levels of *TFAM*, *HSPE1*, and *CYC1* in high-grade serous ovarian cancer were significantly higher than healthy fallopian tube. The results from the TCGA database showed that *HSPE1* and *CYC1* were significantly upregulated in ovarian cancer tissues, but *TFAM* was not statistically significant (Figure 2a–d).

### 4.3 Protein expression of candidate genes in ovarian cancer

**Figure 3:**
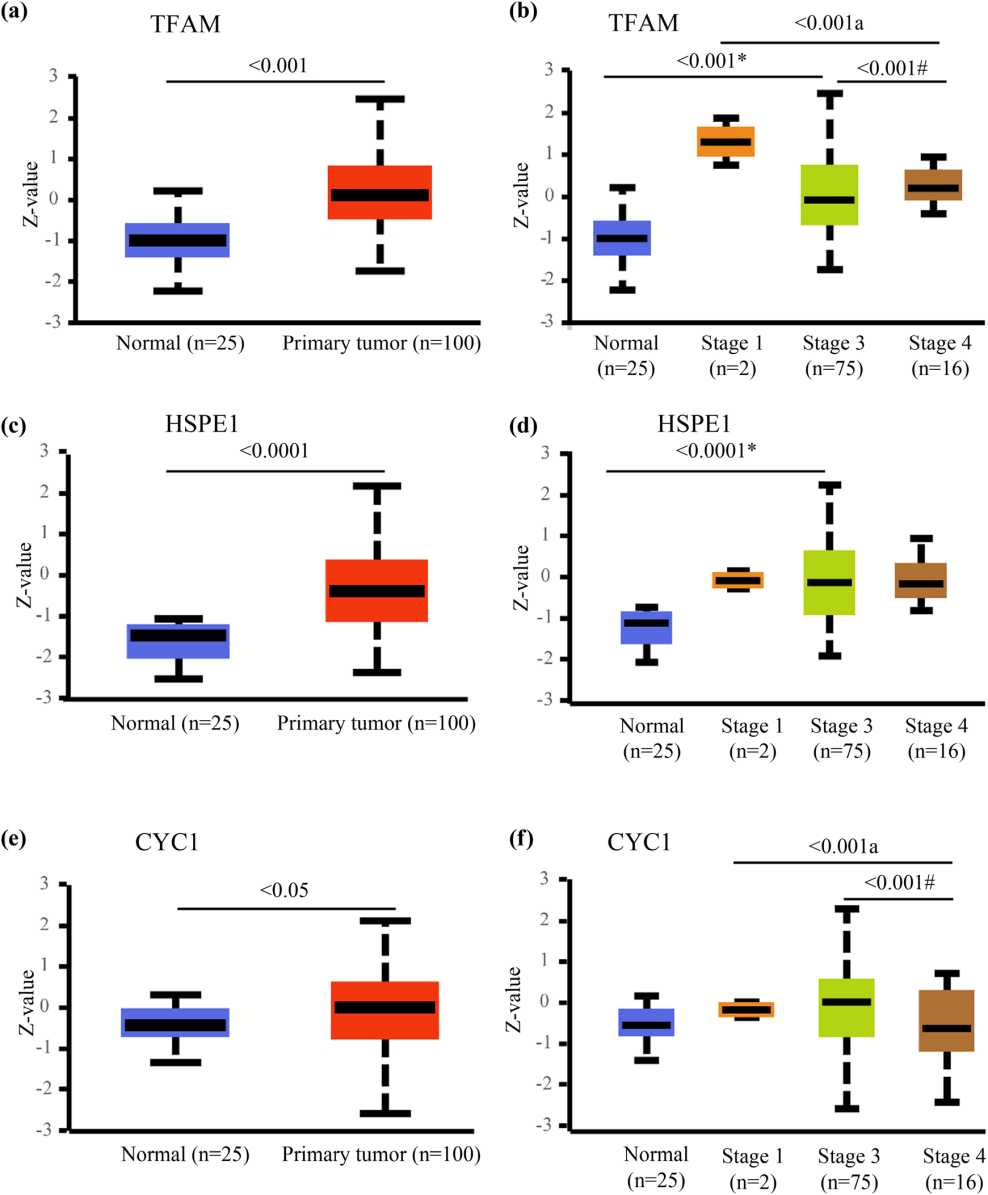
Protein expression levels of candidate genes in ovarian cancer. (a), (c), and (e) Comparison of the protein levels of TFAM, HSPE1, and CYC1, respectively, between the ovarian cancer tissue and the normal tissue. (b), (d), and (f) Protein levels of HSPE1, HSPE1, and CYC1, respectively, in normal tissue and ovarian cancer tissues of different clinical stages. (*) Comparison between stage III and normal. (#) Comparison between stage III and stage IV. (a) Comparison between stage I and stage IV.

Next, we used the CPTAC platform in the UALCAN (The University of ALabama at Birmingham CANcer data analysis Portal) database to query the protein levels of candidate genes in ovarian cancer and normal tissues. The protein levels of TFAM, HSPE1, and CYC1 were significantly increased in ovarian cancer tissues compared with normal tissues (Figure 3a, c, and e). Furthermore, the protein levels of TFAM, HSPE1, and CYC1 varied according to clinical stages of ovarian cancer. Although the TFAM and HSPE1 total protein expression levels of ovarian cancer tissues of each clinical stage were higher than those of normal tissues, the difference was only significant between normal and stage 3 tissues. The protein expression level of TFAM in ovarian cancer tissues in stage III was slightly lower than that in stage I and stage IV, but there was no significant difference between stage III and stage I. The protein level of TFAM in stage IV ovarian cancer was significantly higher than that in stage III (Figure 3b). Only in stage III ovarian cancer tissues was the HSPE1 protein expression level significantly higher than that in normal tissues, but there was no significant difference between stage III, stage IV, and stage I (Figure 3d). The protein level of CYC1 in stage I, III, and IV ovarian cancer tissues was not significantly different from that in normal tissues (Figure 3f).

### 4.4 Effect of expression levels of candidate genes on OS of ovarian cancer

**Figure 4:**
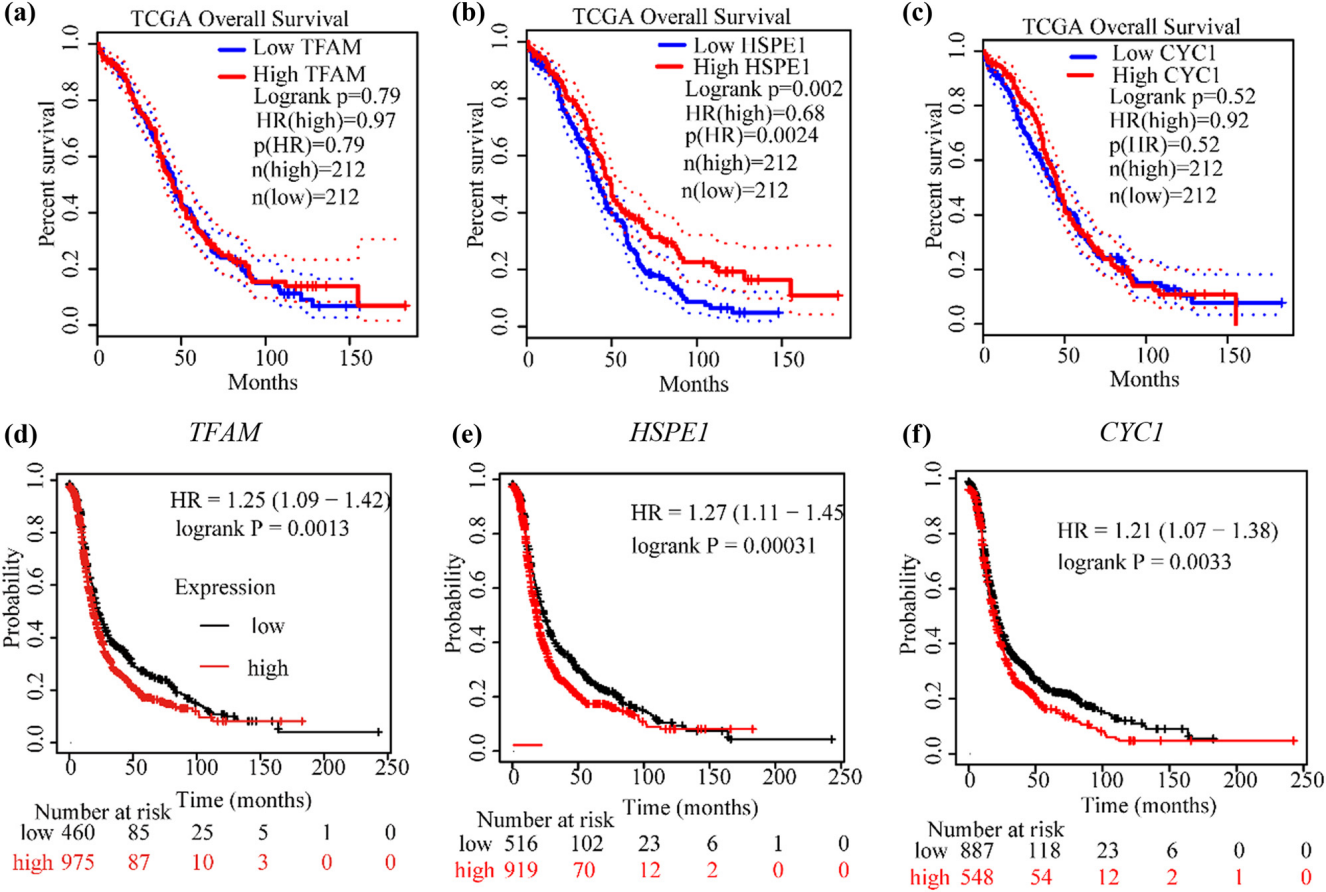
Effect of the expression levels of candidate genes on the OS of ovarian cancer patients. (a)–(c) OS data from the GEPIA 2 database. (d)–(f) OS data from the Kaplan‒Meier plotter database.

Using the Kaplan‒Meier plotter database (gene expression data and relapse-free and OS information downloaded from GEO, EGA, and TCGA) and GEPIA 2 database (gene expression data and relapse-free and OS information were downloaded from the TCGA), we examined the OS of patients based on high and low expression of *TFAM*, *HSPE1*, and *CYC1* in ovarian cancer. High and low expression levels were determined based on the median expression values of the genes in the dataset. The GEPIA 2 database results showed that low expression of *HSPE1* was associated with a poor prognosis, but there was no association between *TFAM* and *CYC1* mRNA expression levels and survival (Figure 4a–c). The Kaplan‒Meier plotter database results showed that high expression of *TFAM*, *HSPE1*, and *CYC1* was associated with an overall poor prognosis in ovarian cancer (Figure 4d–f).

### 4.5 Expression status of TFAM in the EOC study population

**Figure 5:**
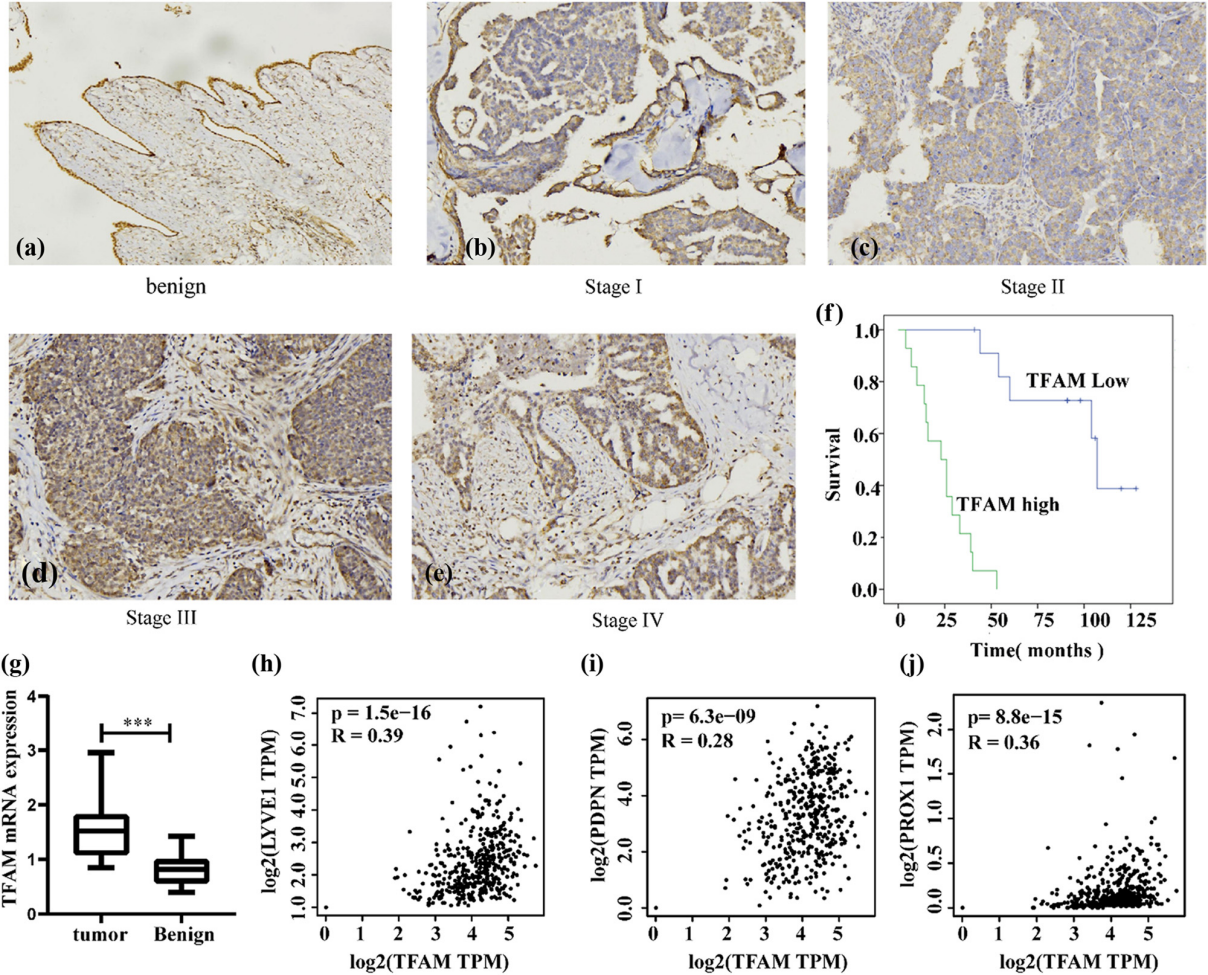
Association between TFAM expression and EOC prognosis and lymphatic endothelial cell marker expression. (a)–(e) TFAM protein expression in benign and EOC tissues at different stages. (f) Kaplan–Meier survival curves for TFAM expression in our EOC patients. (g) Expression of TFAM. (h)–(j) TFAM gene expression correlation with lymphatic endothelial cell marker genes. *** *P* < 0.001.

To investigate the relationship between TFAM expression and the clinicopathological features of EOC patients, immunohistochemistry was performed on primary EOC specimens and benign tissue. Strong positive TFAM staining was mainly observed in the cytoplasm (Figure 5): TFAM was positively expressed in 64.58% (31/48) of the EOC tissues, and the TFAM staining rate in benign ovarian tissues was 5.26% (2/38). There was a significant difference between the positive rate of *TFAM* in EOC and benign ovarian tumors (*χ* ^2^ = 9.465, *P* < 0.0001; S1 File).

Clinicopathological analysis of 48 EOC patients showed that TFAM expression was significantly associated with tumor clinical stage (*P* = 0.004) and lymphatic metastasis (*P* = 0.043). There were no significant correlations between TFAM expression and the remaining clinicopathological features, including age and differentiation (*P* > 0.05) (Table 3). Importantly, concerning OS, positive TFAM expression correlated with a worse OS rate than negative TFAM expression (Figure 5f). The TFAM mRNA expression level was further measured by qRT-PCR. The results showed that *TFAM* mRNA expression in EOC tissues was significantly higher than that in benign tissues (Figure 5g). Given that TFAM expression is associated with lymph node metastasis, we used the GEPIA2 database to search for associations between the expression level of TFAM and the expression levels of the lymphatic endothelial cell markers LYVE1, PDPN, and PROX1 [26,27] in ovarian cancer tissue, and the results showed that the mRNA expression level of *TFAM* was positively associated with that of LYVE1, PDPN, and PROX1 (Figure 5h–j).

**Table 3:** Relationship between TFAM protein expression and clinicopathological parameters of ovarian cancer patients (*n*, %)

		*n*	TFAM		*χ* ^2^	*P*
Clinicopathological parameters			−	+		
Age (years)					0.006	0.895
	<50	17	3 (17.65)	14 (82.35)		
	≥50	31	7 (22.58)	24 (77.42)		
Stage					9.340	0.004**
	I–Ⅱ	15	8 (53.33)	7 (46.67)		
	Ⅲ–Ⅳ	33	13 (39.39)	20 (60.60)		
Differentiation					0.090	0.431
	Low and moderate	26	11 (42.31)	15 (57.69)		
	High	22	11 (50.00)	11 (50.00)		
Lymphatic metastasis					3.320	0.043*
	−	15	7 (46.67)	8 (53.33)		
	+	33	14 (42.42)	19 (57.57)		

Notes: **P < 0.01 and *P < 0.05.

### 4.6 Knockdown of TFAM induces EOC cell apoptosis and inhibits cell proliferation *in vitro*

**Figure 6:**
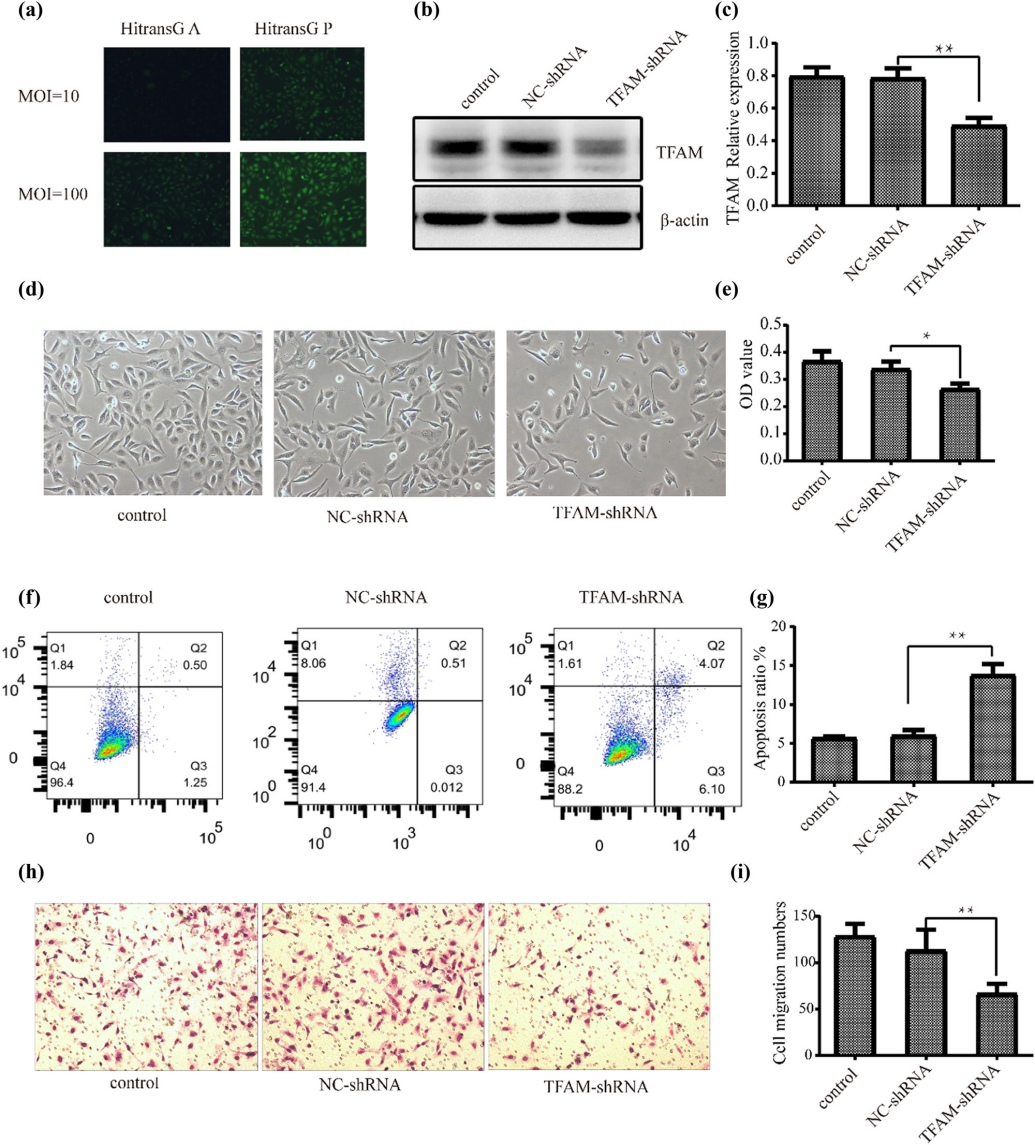
Knockdown of *TFAM* induces EOC cell apoptosis and inhibits cell proliferation *in vitro*. (a) Screening of optimal infection conditions for lentiviral vectors. (b) and (c) Silencing effect of *TFAM* was verified by western blotting (*n* = 3). (d) Cell morphology of SK-OV-3 cells observed under an inverted microscope (100×). (e) Cell proliferation of SK-OV-3 cells by CCK-8 assay (*n* = 5). (f) and (g) Cell apoptosis detected by flow cytometry (*n* = 3). (h) and (i) Cell migration Transwell assay (*n* = 3). **P < 0.01, *P < 0.05.

**Figure 7:**
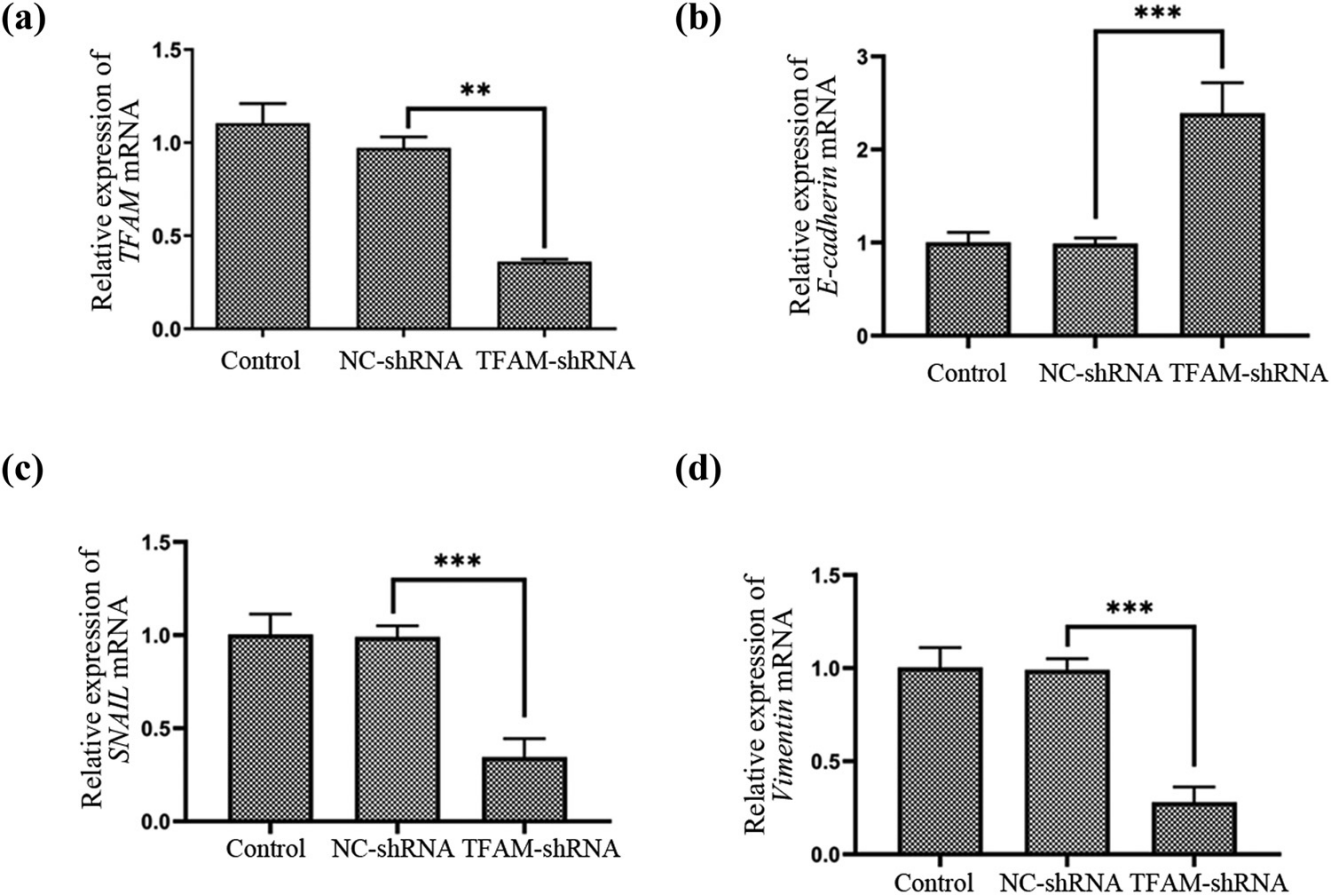
Validation of TFAM shRNA silencing efficiency and mRNA expression level of EMT-related genes. (a) Efficiency TFAM mRNA silencing was verified by qRT-PCR (*n* = 3). (b) Relative expression of E-cadherin mRNA (*n* = 3). (c) Relative expression of SNAIL mRNA and (d) Relative expression of vimentin mRNA (*n* = 3). ***P < 0.001, **P < 0.01.

We next transfected SK-OV-3 cells with the *TFAM*-shRNA lentiviral vector and the negative control lentiviral vector. The optimal transfection conditions were determined by the number of cells expressing green fluorescent protein fluorescence. The MOI value was 100, and transfection for 72 h with HitransG P solution was the best infection condition for follow-up experiments (Figure 6a). The transfection efficiency was confirmed both by qRT-PCR (Figure 7a) and western blotting (Figure 6b and c; for unadjusted images of all blots refer S2 File). Cell morphology was observed under an inverted microscope. The results showed that the cells were dominated by spindle cells with absent intercellular connections, and the number of cells decreased significantly (*P* < 0.05) after *TFAM* knockdown (Figure 6d). Subsequently, the results of the CCK-8 assay demonstrated that *TFAM* knockdown significantly (*P* < 0.05) inhibited cell proliferation (Figure 6e). In addition, apoptosis was further confirmed by flow cytometry. As shown in Figure 6f and g, the apoptotic cell percentage increased significantly with *TFAM* knockdown.

### 4.7 Knockdown of TFAM inhibits EOC cell migration and EMT *in vitro*

**Figure 8:**
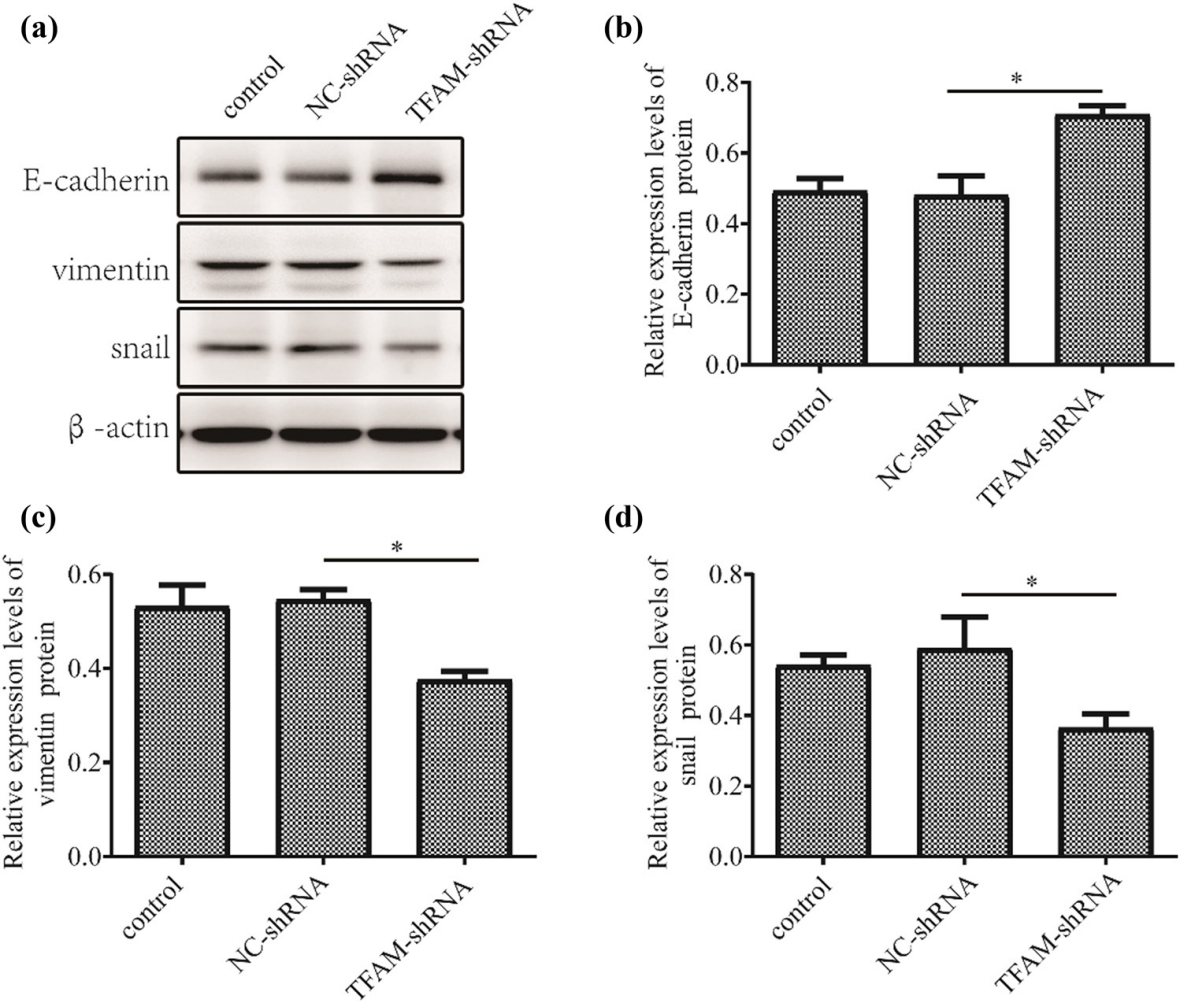
TFAM inhibits EOC cell EMT *in vitro*. Western blotting (a) and quantitative analysis (b)–(d) of the expression of EMT markers (*n*  =  3). *P < 0.05.

To clarify the relationship between *TFAM* and EOC metastasis and EMT, in the second part of the experiment, we mainly explored the influence of *TFAM* on cell metastasis and EMT in ovarian cancer. Moreover, we observed that cell migration was suppressed in SK-OV-3 cells transfected with *TFAM*-shRNA compared with cells transfected with NC-shRNA using a Transwell assay. Consistently, the Transwell migration assay also revealed that *TFAM* knockdown significantly decreased the number of migrated cells (Figure 6h and i). Consistent with the above findings, *TFAM* knockdown markedly decreased the expression of the EMT markers *Vimentin* and *Snail*; in contrast, the expression of *E-cadherin* was increased (Figures 7b–d and 8a–d). These results indicate the potential carcinogenic role of *TFAM* in EOC.

### 4.8 TFAM enhances lymphangiogenesis by increasing the expression levels of VEGF-A, VEGF-C, and VEGF-D

**Figure 9:**
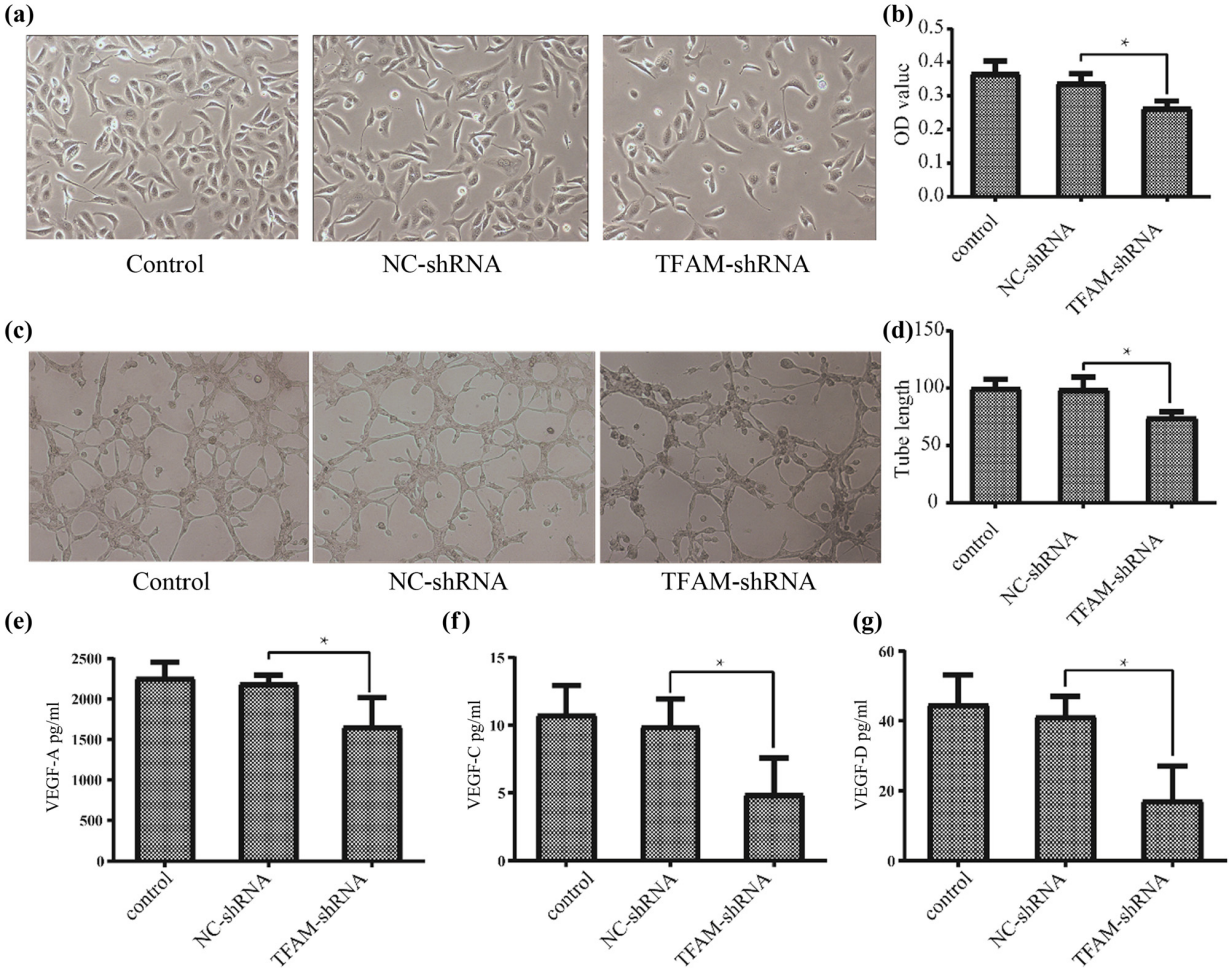
*TFAM* enhanced lymphangiogenesis by increasing the expression levels of VEGF-A, VEGF-C, and VEGF-D. (a) Cell morphology of HLECs observed under an inverted microscope (100×). (b) Cell proliferation of HLECs by CCK-8 assay (*n* = 5). (c) and (d) Tube formation of HLECs observed under an inverted microscope (100×) (*n* = 3). (e)–(g) Concentration of VEGF-A, VEGF-C, and VEGF-D in SKOV-3 cellular supernatant detected by ELISA (*n* = 3). *P < 0.05.

To further examine the effect of *TFAM* on lymphangiogenesis, HLECs were cultured with CM from *TFAM* knockdown SK-OV-3 cells as described previously [24]. The results showed that HLECs cultured with CM from *TFAM* knockdown SK-OV-3 cells exhibited markedly decreased proliferation and tube formation (Figure 9a–d). VEGF-A, VEGF-C, and VEGF-D have been reported to mediate tumor lymphangiogenesis in many human cancers. However, the effect of *TFAM* on VEGF-A, VEGF-C, and VEGF-D expression in cancer has not been well explored. Therefore, we measured VEGF-A, VEGF-C, and VEGF-D protein levels in *TFAM* knockdown SK-OV-3 cell supernatants by ELISA and their mRNA levels in *TFAM* knockdown SK-OV-3 cells. We found that knockdown of *TFAM* significantly reduced VEGF-A, VEGF-C, and VEGF-D protein expression (Figure 9e–g) and mRNA expression (S3 File).

## 5 Discussion

This study of several large cohorts from the TCGA and GEO databases demonstrates that elevated TFAM correlates significantly with metastasis and poor EOC patient survival. We validated that TFAM facilitates the motility of EOC cells through EMT and is correlated with lymphangiogenesis. Thus, TFAM may be a new mitochondria-related biomarker of metastasis and prognosis and is expected to be a candidate therapeutic target for EOC.

Mitochondria play a central and multifunctional role in malignant tumor progression, and targeting mitochondria provides therapeutic opportunities [21]. Mitochondria play a major role in cancer progression and can influence all processes linked to oncogenesis, from malignant transformation to metastatic dissemination [22–24]. Here, we examined mitochondrial genes in benign tissue and in high-grade EOC and performed exploratory analyses of biological behavior.

First, analysis of EOC RNA-seq data (GSE209964) from the GEO database showed that the mitochondrial genes *TFAM*, *HSPE1*, and *CYC1* were significantly upregulated in high-grade serous ovarian cancer. In the next step, we confirmed the protein expression of *TFAM*, *HSPE1*, and *CYC1* in the CPTAC database. We identified mitochondrial function-related genes *TFAM*, *HSPE1*, and *CYC1* via screening of high-throughput data in GEO database. We further investigated the mRNA expression of these three genes in the TCGA database and assessed their protein levels in the UALCAN database. Analysis of the TCGA database did not show a significant difference in *TFAM* mRNA expression between tumor tissue and fallopian tube tissue (Figure 2), and the analysis based on the UALCAN database did not show significant differences among different stages of ovarian cancer in terms of CYC1 protein level (Figure 3). Because we believe that the final effect is exerted by proteins, we first selected *TFAM* and *HSPE1* as candidate genes due to the significant differences in the levels of their encoded proteins (Figure 3). However, the survival analyses based on *HSPE1* expression in the TCGA database and Kaplan‒Meier plotter database yielded opposite results, and both correlations were statistically significant according to the *P*-values (Figure 4); this might be caused by the difference in sample size between the TCGA database and the Kaplan–Meier plotter database. Therefore, after comprehensive consideration, we ultimately chose the *TFAM* gene as the target gene for subsequent research and explored its role in the occurrence and development of ovarian cancer. *TFAM* plays a pivotal role in the regulation of mitochondrial biogenesis and the TCA cycle [25]. *TFAM* is involved in tumor metastasis in breast cancer [26], and its protein levels are elevated in high-grade serous ovarian cancer tumor biopsies (according to the reference value) [27]. However, the role of TFAM in the lymphatic metastasis of ovarian carcinomas remains to be further explored. Several studies have shown that TFAM is abnormally expressed in various tumor tissues, and it is related to lymph node metastasis and tumor malignancy degree [28–31]. Studies on TFAM and ovarian cancer show that TFAM is also highly expressed in ovarian cancer [32–35]. Our clinicopathological analysis results showed that TFAM expression was significantly associated with tumor clinical stage and lymphatic metastasis. Importantly, TFAM-positive expression correlated with a worse OS rate. However, the mechanism of TFAM in ovarian cancer has not been thoroughly studied.

Uncontrolled continuous proliferation is one of the most basic biological characteristics of all malignant tumors, and clarifying the mechanism of abnormal tumor cell proliferation has important clinical transformation significance. Several studies have shown that TFAM is involved in cell proliferation, invasion, and apoptosis [36–38]. We showed that silencing TFAM can inhibit cell proliferation and promote apoptosis in ovarian cancer cells. Therefore, we speculate that TFAM has the potential to become a drug target for the treatment of ovarian cancer.

EMT is related not only to improved motility of cancer cells but also enhanced stem cell characteristics and drug resistance of tumor cells. Therefore, it can drive cancer metastasis, tumor recurrence, and treatment resistance [39]. The expression level of *TFAM* increases during the induction of EMT in breast cancer cells. Our results confirmed that silencing *TFAM* by shRNA decreased the cell metastasis ability. At the same time, we found that the expression levels of Vimentin and snail proteins decreased, while the expression of E-cadherin protein increased. Downregulating *TFAM* in colorectal cancer can also downregulate *E-cadherin*, *vimentin*, and *snail* proteins. Therefore, we speculate that *TFAM* is involved in ovarian cancer metastasis by regulating tumor cell EMT.

Relevant research reports show that the lymphatic system is the most important mechanism of malignant tumor metastasis [40]. Ovarian cancer is also a malignant tumor with high lymph node metastasis potential, with 70% of EOC patients experiencing pelvic and paraaortic lymph node metastasis [41]. Lymphatic vessels are composed of lymphatic vessel endothelial cells (LECs). As a special subgroup of endothelial cells, LECs have the ability to form lumens [42]. Compared with vascular endothelial cells, LECs have large gaps, incomplete basement membrane between cell layers, and low lumen pressure, which makes it easy for tumor cells to enter the lumen through the walls of lymphatic vessels and achieve lymph node metastasis. Studies have revealed that high tumor lymphatic vessel density in ovarian cancer tissue is significantly related to peritoneal metastasis, distant metastasis, and lymph node metastasis [43], but lymph node metastasis is a complex process, and its molecular mechanism has not yet been fully clarified. Lymphatic vessel formation involves the regulation of a variety of promoting and inhibitory factors. *VEGF-A*, *VEGF-C*, and *VEGF-D* are the most studied cytokines at present, and *VEGF-C* is currently recognized as a cytokine that plays a key role in lymphatic vessels [43]. Relevant studies have shown that *VEGF-C* and *VEGF-D* specifically bind to the receptor *VEGFR3* in endothelial cells and activate its downstream signaling pathway to induce the formation of many lymphatic vessel buds toward tumor cells, causing lymphatic vessels to expand into tumor tissue. Second, *VEGF-C* and *VEGF-D* combine with *VEGFR3*, leading to its phosphorylation and promoting the proliferation of LECs [44–46]. In our first study, we found that the high expression of *TFAM* in EOC tissue was significantly correlated with lymph node metastasis, which indicates that *TFAM* may promote lymphangiogenesis in EOC. Our results show that silencing *TFAM* in ECO cells reduced the *VEGF-A*, *VEGF-C*, and *VEGF-D* contents in the supernatant. The proliferation, metastasis, and tube formation of HLECs are the main processes underlying lymphangiogenesis [47]. In this study, the stimulation of HLECs with CM from *TFAM* knockdown SK-OV-3 cells markedly decreased LEC proliferation and tube formation. This indicates that *TFAM* is involved in the lymphatic metastasis of ovarian cancer cells and can be used as a therapeutic target for lymphatic metastasis of ovarian cancer.

However, this study has some limitations. First, this study was a single-center study, and the number of involved participants was small. Second, the analysis of the EOC transcriptome sequencing dataset GSE209964 from the GEO database showed that *TFAM* was one of the three candidate genes related to EOC, which was further confirmed by qRT-PCR of the samples collected in this study. However, this finding was contradicted by the TCGA database analysis, which indicated that *TFAM* expression was not significantly different between ovarian cancer tissue and benign tissue. Therefore, to validate the results of this study, a multicenter study with a larger sample size remains necessary. Third, this study lacked normal ovarian tissue in the control group. While benign lesions provide useful insights into non-malignant conditions, the inclusion of normal ovarian tissue would offer a more comprehensive baseline for understanding gene expression. Future studies that incorporate normal tissue remain to be carried out to strengthen the comparative analysis. Fourth, only one EOC cell line was used in the cytological experiments, which might also cause bias in the final results. Therefore, experiments involving more cell lines should be performed in the future to further verify the findings of this study. Fifth, we did not validate the association between *TFAM* and the lymphatic endothelial cell markers in EOC samples used in this study due to insufficient funding, and this limitation might cause bias to the outcomes of this study. Such analyses are worthwhile and should be performed with a large sample size in the future. Last, the analysis of the association between the protein level of TFAM and the clinical stage of ovarian cancer based on CPTAC platform had a small sample size, particularly for the analysis involving stage I samples (*n* = 2), which might cause bias to the outcome obtained in this study.

*TFAM* is highly expressed in EOC tissues, and its expression level might be significantly correlated with clinical stage and lymph node metastasis. The survival period of TFAM-positive patients is shorter than that of TFAM-negative patients; silencing *TFAM* in EOC cells can inhibit cell proliferation and migration ability and induce cell apoptosis. TFAM may promote the secretion of VEGF-A, VEGF-C, VEGF-D, and other cytokines in EOC cells, promote the formation of new lymphatic vessels, and lead to lymph node metastasis of EOC cells. Our results provide new insights into the biological functions and potential mechanisms of TFAM in EOC and provide new therapeutic targets for the diagnosis and treatment of EOC.
